# Subcellular localization of the P2X4 receptor in sensory hair cells of Wistar rat cochlea

**DOI:** 10.1007/s00418-025-02386-1

**Published:** 2025-05-20

**Authors:** Ziyin Huang, Jacqueline M. Ross, Shelly C. Y. Lin, Prakansha N. Kumar, Kevin Roy, Srdjan M. Vlajkovic, Peter R. Thorne, Haruna Suzuki-Kerr

**Affiliations:** 1 Department of Physiology, The University of Auckland, Building 502-401, 85 Park Road, Grafton, Auckland, 1023 New Zealand; 2 Section of Audiology, The University of Auckland, Auckland, New Zealand; 3 Eisdell Moore Centre, The University of Auckland, Auckland, New Zealand; 4 Department of Anatomy and Medical Imaging, The University of Auckland, Auckland, New Zealand; 5 Biomedical Imaging Research Unit, The University of Auckland, Auckland, New Zealand

**Keywords:** Cochlea, Sensory hair cell, Purinergic signalling, P2X receptor, P2X4, ATP, Organ of Corti

## Abstract

Our sense of hearing starts in the inner ear organ, the cochlea, which contains two types of auditory hair cells for signal transduction. Earlier research showed that the complex cochlear physiology is regulated in part by purinergic signalling through activation of purine-mediated P2X, P2Y and adenosine receptors expressed in the cochlea. This study aims to extend our knowledge of purinergic signalling in the cochlea by comprehensively characterising the expression of the P2X4 receptor subtype. Wistar rat cochlea (embryonic day 20.5–6 weeks, both sexes) were collected, and the P2X4 expression was examined by immunohistochemistry. Robust P2X4 expression was found in the organ of Corti (OoC) in the inner hair cells (IHCs) and outer hair cells (OHCs), confirmed by double-labelling with hair cells (HCs) marker myosin VIIa. In IHCs, a robust cytoplasmic P2X4 expression occurred throughout the cell body, with the most intense signal at the medial side. In OHCs, P2X4 formed puncta near the apical and basal ends of the cell body. Using markers for subcellular organelles, P2X4 immunoreactivity was predominately associated with the trans-Golgi network apparatus and early endosomes in IHC and with early endosomes and lysosomes in OHC in the mature cochlea. In both cell types, some co-localisation of P2X4 with presynaptic marker was also observed. Taken together, these observations suggest unique roles for P2X4 in mature IHCs and OHCs as a purinergic receptor subtype responsible for the homeostatic regulation of hair cells and auditory sensory transduction.

**Supplementary Information:**

The online version contains supplementary material available at 10.1007/s00418-025-02386-1.

## Introduction

According to the World Report on Hearing (WHO, 2021), hearing loss affects 1.5 billion people globally, and this number is expected to grow to 2.5 billion by 2050 (WHO, 2021). The majority of cases are sensorineural hearing loss (SNHL), characterised by degenerative changes in the cochlea and the auditory nerve. There are few effective pharmacological treatments for SNHL, and the development of such treatment requires further understanding of cochlear physiology and pathophysiology at the cellular and molecular levels. The organ of Corti (OoC), the sensory apparatus within the cochlea, contains two types of auditory sensory cells, the inner hair cells (IHC) and outer hair cells (OHC). Hair cells (HCs) are so-called owing to the mechanosensory stereocilia located on the apical side of the cell supported by an actin–rich cuticular plate and are essential for sound transduction (Goodyear et al. 2005). Approximately 3500 IHCs are aligned as a single continuous row and 12,000 OHCs arranged in three rows more laterally in the human cochlea. In humans and animal models, the loss of hair cells (HCs), particularly OHC and loss of the synapses and neurons innervating IHC, has been observed as a common underlying pathology associated with SNHL (Liberman and Kujawa 2017; Wu et al. 2019; Wu et al. 2020).

Purinergic signalling is involved in many cellular functions and pathologies in the inner ear and is considered to be a potential therapeutic target for inner ear disorders. Purinergic signalling pathways are activated by extracellular nucleotides adenosine triphosphate (ATP), adenosine diphosphate (ADP), uridine triphosphate (UTP), uridine diphosphate (UDP) and adenosine (Burnstock 1997). There are two classes of purinergic receptors: P1 receptors (A_1_, A_2A_, A_2B_ and A_3_) and P2 receptors (P2X1-7 P2Y1, 2, 4, 6, 11-14) (Burnstock 2007). Many of these purinoceptor subtypes have been identified in the cochlea (Köles et al. 2019; Vlajkovic and Thorne 2022). P2X receptors form trimeric ligand-gated ion channels that are nonselectively permeable to cations (Na^+^, K^+^, Ca^2+^) (Burnstock 2007). Earlier studies had suggested a potential role for P2X receptors in the cochlea, such as the regulation of afferent neuronal activity in response to agonists released in the perilymph (Robertson and Paki 2002). More specific roles for P2X subtypes are emerging on the basis of molecular and functional investigations; P2X2 expressed in cells lining the endolymphatic compartment and in the stereocilia of OHC participates in the reduction of endocochlear potential during sound transduction and in modulating the sound sensitivity, respectively (Jarlebark et al. 2000; Morton-Jones et al. 2015; Wang and Neuhuber 2003). P2X1 and P2X7 localised near synaptic terminals of the auditory neurons on IHCs and in the neurites of the spiral ganglion neuron (SGN) (Nikolic et al. 2001; Nikolic et al. 2003) may play roles in Ca^2+^-dependent uncoupling of synapses known to occur at these postsynaptic terminals (Liberman and Kujawa 2017). P2X3 expressed in the developing spiral ganglion neurons regulates the branching of afferent fibres (Huang et al. 2006; Wang et al. 2020). By contrast, the molecular expression of P2X4-6 has not been clearly demonstrated in the cochlea. P2X4 has several unique features compared with other P2X subtypes. The human monomeric P2X4 channel is sensitive to the extracellular Ca^2+^ concentration, with slow desensitisation compared with P2X1-3 isoforms requiring 4 s at a 30 μM concentration ATP (Hattori and Gouaux 2012). The cytoplasmic expression of P2X4 and localisation to lysosomes have been reported (Huang et al. 2014; Murrell-Lagnado and Frick 2019). Intracellular P2X4 exhibits pH sensitivity (inactivated at low pH) and can be regulated by the pH within the lysosomal lumen (P. Huang et al. 2014; Murrell-Lagnado and Frick 2019). In the guinea pig cochlea, the functional expression of P2X4 has been reported in the endothelial cells of the spiral ligament where it appears to regulate cochlear blood flow (Wu et al. 2011) and in hair cells (Szücs et al. 2004); however, the detailed P2X4 distribution in the cochlea still remains to be characterised. In the vestibular system of the inner ear, P2X4 molecular and functional expression has recently been reported in vestibular supporting cells (Jeong et al. 2020).

In this study, we investigated the distribution of P2X4 in the developing and adult rat cochlea. Expression was confined to OHCs and IHCs and located solely in the cytoplasm. Co-localisation with markers of endoplasmic reticulum, Golgi apparatus and lysosomes suggests that P2X4 is associated with different intracellular organelles (Golgi–endoplasmic reticulum (ER) in IHC and lysosomes in OHC) and may mediate calcium buffering within these intracellular organelles.

## Method and materials

### Animals

The use of animals for this project was approved by the University of Auckland Animal Ethics Committee (AEC, no. AEC002251). All animals were supplied by the Vernon Jensen Unit (VJU; The University of Auckland). Wistar rats of various ages and both sexes were used for this study; embryonic day 20.5, postnatal day 4 (P4), postnatal day 8 (P8), postnatal day 21 (P21), and 6-week-old (adult).

### Tissue preparation

General chemicals were purchased from Thermo Fisher Scientific (Auckland, New Zealand), unless otherwise specified; 4% w/v paraformaldehyde (PFA, pH 7.4) was prepared with 0.1 M phosphate buffer (PB, 24.6 mM NaH_2_PO_4_ and 75.4 mM NaHPO_4_, pH 7.4) for fixation of all samples. P21 and adult rats were first anaesthetised and euthanised by drug overdose (pentobarbital, ProVet NZ Pty Ltd), followed by perfusion of 0.1% w/v NaNO_2_ in phosphate-buffered saline (PBS, pH 7.4, Gibco) and 4% PFA through the left ventricle. The temporal bones were removed from the cranium and a small puncture was carefully made in the round window membrane to aid the penetration of PFA into the cochlea. The cochlea was then immersion-fixed in 4% PFA at room temperature (RT) for 24 h. Cochleae were washed with PBS three times (10 min each). Adult cochlear tissues were decalcified by immersion in 4% w/v ethylenediaminetetraacetic acid (EDTA, in 0.1PB, pH 7.4) at room temperature for up to 2 weeks with regular change of EDTA solution. Cochleae from Wistar rats younger than P8 were removed and immersed in PFA (4% PFA at room temperature (RT)) for 24 h, and further dissection was carried out without decalcification. For the organ of Corti (OoC) whole mount preparations, cochleae were micro-dissected in PBS and segments of OoC approximately equivalent to half turn were taken from the apical, middle and basal turn. For cryosectioning, cochleae were cryoprotected sequentially in 10% and 20% sucrose (w/v in PBS) for 1 h at room temperature, then in 30% sucrose overnight at 4°C followed by embedding in Tissue-Tek Optimal Cutting Temperature Compound (OCT, ProSciTech, Australia) at −80 °C. The tissue was cryosectioned at 20–30 μm (Leica, CM3050S) in the axial plane through the modiolus and cochlear ducts. Microdissected or cryosectioned tissue was stored in PBS for up to 1 week at 4 °C before processing for immunohistochemistry.

### Immunohistochemistry

The list of primary antibodies used in this study and dilutions are summarised in Table 1. A polyclonal primary antibody raised in rabbits against the C-terminal domain of the rat P2X4 subunit (Alomone Inc., Jerusalem, Israel, catalogue no. APR-002) was used for detection of P2X4 by immunohistochemistry. This antibody has been validated using P2X4-knockout animals (Lalisse et al. 2018; Sim et al. 2006; Wyatt et al. 2014). Other antibodies used as cell-type specific markers and organelle markers are summarised in Table 1. Immunohistochemistry was performed following the protocol established previously (Fok et al. 2020; Han et al. 2019). Blocking solutions and antibody diluent solutions were prepared as following; blocking solution for whole mounts (10% v/v normal goat serum (NGS) and 2.5% (v/v) TritonX in PBS), blocking solution for cryosection (10% v/v NGS, 1% v/v TritonX in PBS), antibody diluent for wholemount (5% v/v NGS and 0.25% v/v TritonX in PBS) and antibody diluent for cryosection (5% v/v NGS and 0.1% v/v TritonX in PBS). Tissues were incubated for 2 h in a blocking solution at room temperature. Tissues were then incubated in the diluted primary antibody overnight at 4 °C. For the anti-P2X4 antibody control, the pre-absorbing peptide was added to the primary antibody solution in a 1mg:1mg ratio and sections or whole mounts were incubated for 2 h following the manufacturer’s protocol (Alomone Labs, Israel). Tissues were washed four times at 1-, 10-, 15- and 30-min intervals in PBS at RT followed by incubation with secondary antibodies overnight at 4 °C in the dark. Secondary antibodies used were goat anti-rabbit immunoglobulin (Ig)G Alexa Fluor 594, goat anti-rabbit IgG Alexa Fluor 488 and goat anti-mouse IgG Alexa Fluor 647 (Thermo Fisher Scientific, all used at 1:500 dilution in antibody diluent). The non-antibody labelling reagents wheat germ agglutinin (WGA) and phalloidin (Table 1) were included in the same mixture with the secondary antibody. From this step forward, tissues were covered to minimise light exposure. After incubation with secondary antibodies, the tissues were washed four times in PBS, incubated in 4′,6-diamidino-2-phenylindole (DAPI, diluted in PBS, 0.02 μg/ml) for 1 h at room temperature, washed four times in PBS and mounted with coverslips on slides with CitiFluor AF1 mountant solution (Agar Scientific Ltd, UK). Slides were stored at 4 °C in the dark until imaging.

**Table 1:** Antibodies used in this study. Relevant information was gathered from datasheets for each antibody

Antibody	Company (catalogue no.)	Epitope	Dilution	Reactivity	Marker for	References
Anti-P2X4, rabbit IgG polyclonal	Alomone (APR-002)	C-terminus of mouse P2X4 isoform	1:1000	Mouse, rat and human	P2X4	(Lalisse et al. 2018), (Sim et al. 2006), (Wyatt et al. 2014)
Anti-SOX2, mouse IgG monoclonal	Santa Cruz Biotechnology Inc. (Sc-365823)	Human SOX2 amino acids 170–201	1:100	Mouse, rat and human	Cochlear supporting cell nucleus	(Oesterle et al. 2007), (Smeti et al. 2011)
Anti-myosin VIIa, mouse IgG monoclonal	Santa Cruz Biotechnology Inc. (sc-74516)	N-terminus of human myosin VIIa	1:50	Mouse, rat and human	Inner hair cells and outer hair cells	(Xiong et al. 2019), (Jung et al. 2019)
Anti-LAMP-1, mouse IgG monoclonal	Santa Cruz Biotechnology Inc. (sc-20011)	Adherent spleen cells of human origin	1:200	Mouse, rat and human	Lysosomes	(Spangenberg et al., 2019), (Oh et al. 2020)
Anti-EEA-1, mouse IgG monoclonal	Santa Cruz Biotechnology Inc. (sc-137130)	N-terminus of human EEA1	1:200	Mouse, rat, human and monkey	Endosomes	(Kuszczyk et al. 2013), (Men et al. 2019)
Anti-GM130, mouse IgG monoclonal	BD Biosciences (610,822)	Rat GM130 aa. 869–982	1:200	Human, dog and mouse	Golgi	(Dandoy-Dron et al. 2003), (Zheng et al. 2010)
Anti-TOM20, mouse IgG monoclonal	Santa Cruz Biotechnology Inc. (sc-17764)	Human Tom20	1:200	Mouse, rat and human	Mitochondria	(Balaker et al. 2013), (Xiong et al. 2019)
Anti-Ctbp2, mouse IgG1 monoclonal	BD Bioscience (612,044)	Ctbp2	1:500	Mouse, rat, human and guinea pig	Pre-synaptic terminal	(Hickman et al. 2018; Kujawa and Liberman 2009; Liberman et al. 2011)
WGA Alexa 647 conjugate	Thermo Fisher (W32466)	Wheat germ agglutinin (WGA)	1:300	All	Plasma membrane	(Graveleau et al., 2005), (Cui et al., 2020)
Phalloidin Alexa 488	Thermo Fisher (A12379)	Phalloidin	1:500	All	Cytoskeletal actin	

### Confocal microscopy

Fluorescently immunolabelled slides were imaged using a Zeiss LSM 800 Airyscan confocal microscope (Carl Zeiss GmbH, Jena, Germany) in the Biomedical Imaging Research Unit (BIRU) at the University of Auckland. Objective lenses used were 10×/0.45 NA Plan Apochromat, 20×/0.8 NA Plan Apochromat and 63×/1.4 NA Plan Apochromat oil immersion. Images were acquired at a pixel resolution of 0.18 μm/pixel for 20×, and 0.035 μm/pixel for 63× in Airyscan mode. The Z series were obtained using a 63×/1.4 NA oil immersion objective lens with a 0.5 μm step size between optical sections. The top limit for the Z series was set at the level of the tip of the stereocilia of the hair cells, as visualised with phalloidin representing the most apical end of the cell body, and the bottom limit was set at the opposite end of the hair cell body at the position where the P2X4 signal had just disappeared from the HCs. A typical Z-stack was 40 μm thick. All of the images were acquired using ZEN 2.6 software (Carl Zeiss, Germany) and exported to TIFF as required for figure preparation or analysis.

### Image analysis and processing

ImageJ (Schneider et al., 2012) was used for the particle analysis and quantification of P2X4 immunolabelling on individual cells from the Z series images (see Supplementary Material for details on methodology). ImageJ ‘line plot profile’ analysis was performed to quantify the relative signal intensity within OHC and IHC in the apical to basal (A-B) direction or the medial to lateral (M-L) direction at nine different locations in each hair cell. Signal intensity along the direction of interest were averaged between nine line profiles. The single average greyscale value for the whole cell was calculated and was used to normalise the line profile (Figs. 3 and 4 and Supplementary Material 2). For co-localisation analysis, the Just Another Co-localisation Plugin (JACoP) plugin in ImageJ was used. In brief, individual channels were separately processed by background subtraction and images were cropped so that the region of interest typically contained eight cells (Supplementary Material 3). JACoP automatically calculates Manders’ co-localisation coefficients and Pearson’s correlation coefficient (Bolte and Cordelières 2006; Dunn et al. 2011). The results were displayed as M1 and M2, each with the value range between 0 and 1.0, where M1 is defined as the ratio of the ‘summed intensities of pixels from the green channel for which the intensity in the red channel is above zero’ to the ‘total intensity in the green channel’. M2 is identified as the same as the red and green reversed. High M1 and M2 coefficients indicate that a large proportion of one signal co-occurs with the other signal. Co-localisation analyses were conducted for each subcellular marker separately, with three cochleae for each marker, and the mean and standard error of the mean (SEM) were calculated. Imaging processing was performed using Adobe Photoshop CC (version 19.1.3, Adobe system Incorporated) to prepare figures. Areas for Airyscan high resolution imaging and analyses were always chosen from the mid-turn of the cochlea with consistent labelling and good morphological preservation.

## Results

### P2X4 expression in the cochlea

**Fig. 1:**
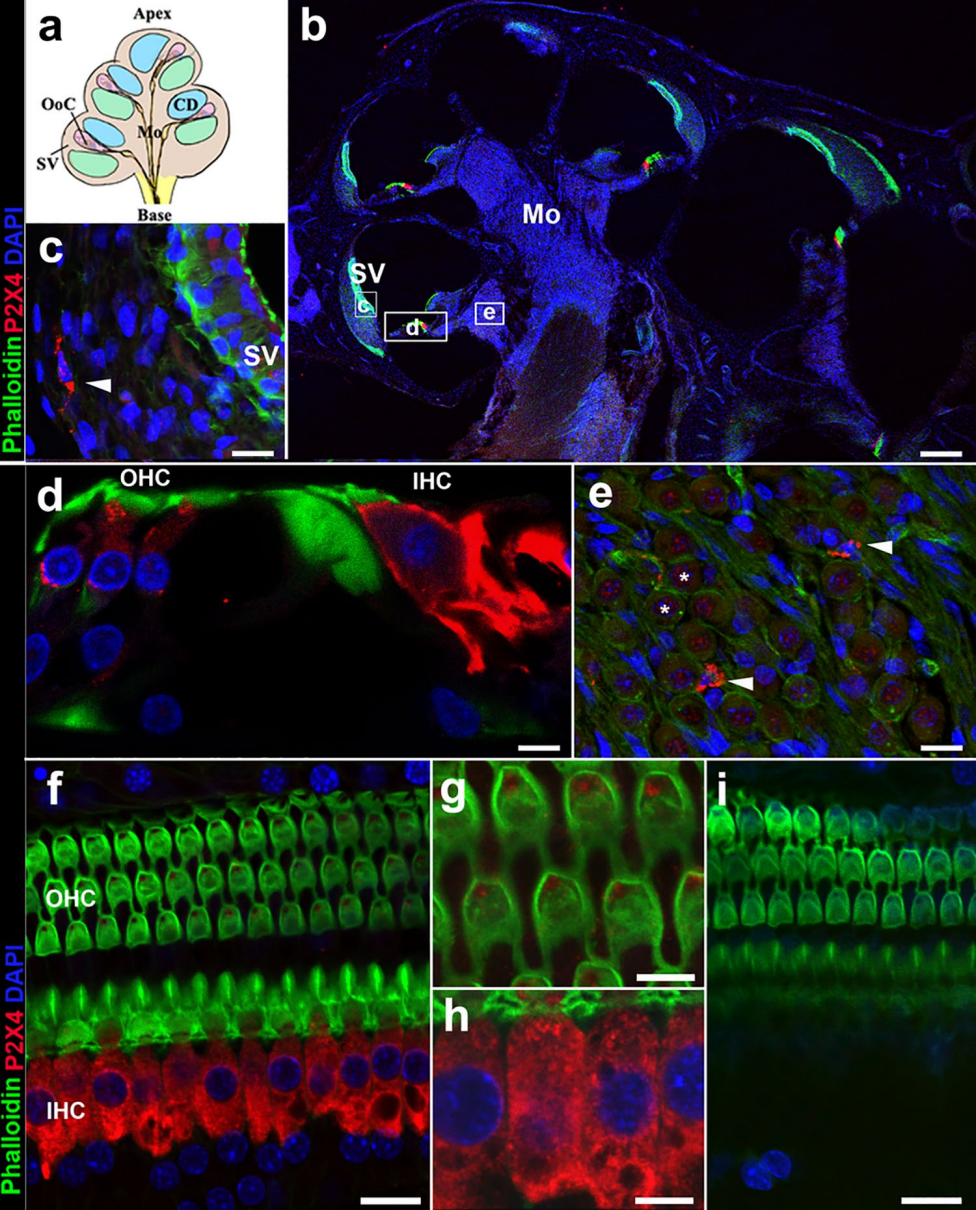
Expression of P2X4 in the adult rat cochlea. **a** Schematic drawing of OoC cryosection; **b**–**i** cryosections (**b**–**e**) and OoC wholemounts (**f**–**i**) were prepared from adult Wistar rat cochlea and labelled with anti-P2X4 antibody (red), phalloidin (green) and DAPI (blue). P2X4 antibodies pre-absorbed with molar excess control peptide (**i**). Representative image from *n* = 6 cochlea. Scale bar 200 µm (**b**), 20 µm (**c**, **e**, **f**, **i**), and 10 µm (**d**, **g**, **h**). *Indicates the spiral ganglion neurons (**e**)

**Fig. 2:**
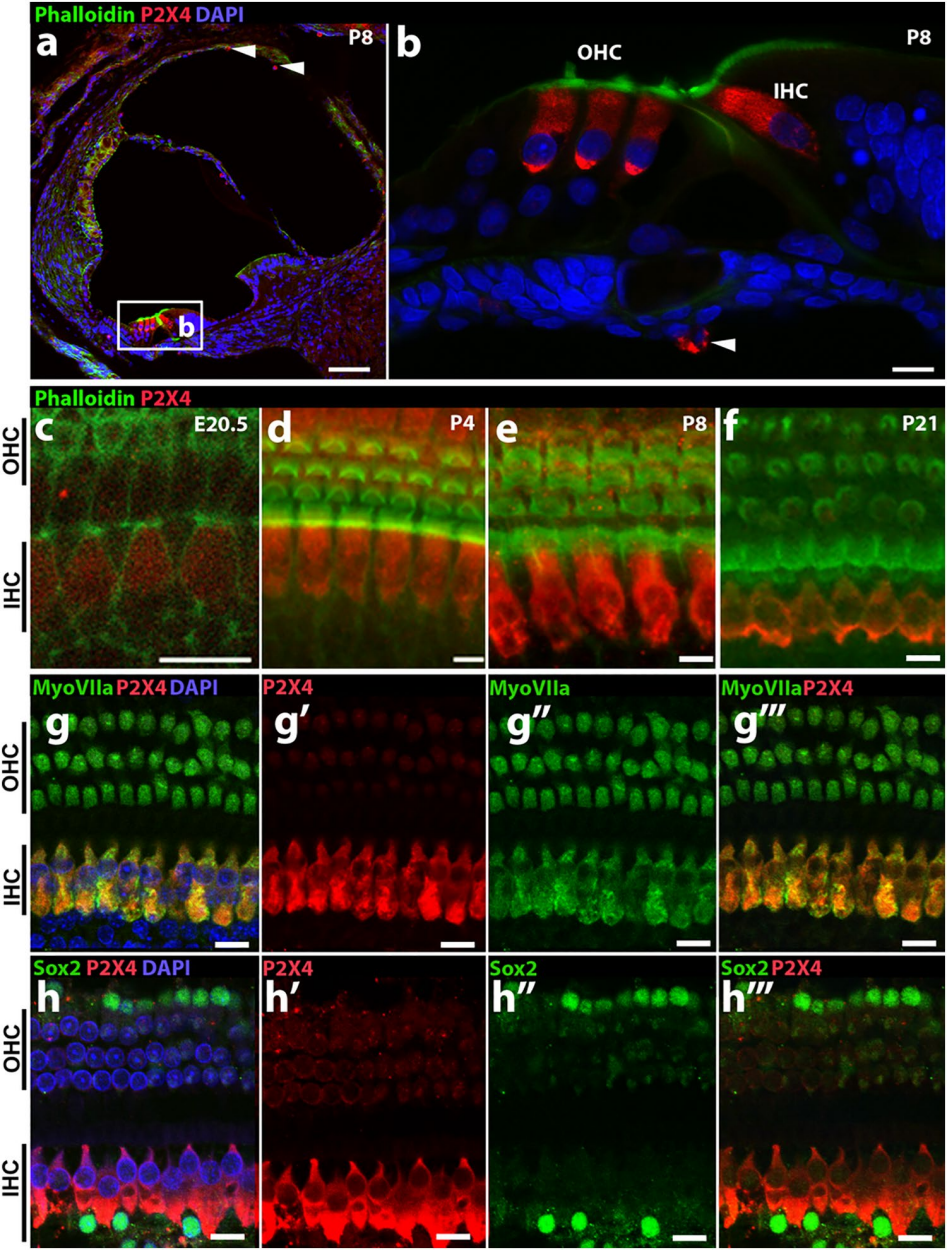
Developmental expression of P2X4 in IHCs and OHCs. **a**–**f** Cryosection (**a**, **b**) and OoC whole mount preparation (**c** - **f**) of Wistar rat cochleae at P8 (**a**, **b**, **e**), E20.5 (**c**), P4 (**d**) and P21 (**f**) labelled with anti-P2X4 antibody (red) and phalloiding (green). **g–g'''**, **h–h'''** Adult cochlea whole mounts were labelled with anti-P2X4 (red) and co-labelled with anti-myosin VIIa (**g–g'''**, green) or anti-SOX2 (**h–h'''**, green) antibodies and DAPI (blue) in the OoC of the adult rat cochlea. Scale bar 50 µm (**a**) and 10 µm (**b**–**h**)

After testing the dilution range 1:50–1:2000, 1:1000 dilution was chosen to have the best signal to background ratio (data not shown) for the anti-P2X4 antibody (Fig. 1). High levels of expression of P2X4 in the rat OoC were observed (Fig. 1d, f). While there was minimal expression of P2X4 in the spiral ligament, some cells resembling fibrocytes showed positive labelling (Fig. 1c, arrowhead). Similarly, while spiral ganglion neurons did not show above background P2X4 labelling (Fig. 1e, asterisks), smaller cells were positively labelled for P2X4 (Fig. 1e, arrowhead). In the OoC, there was a relatively higher expression in IHC and, to a lesser extent, in OHCs (Fig. 1d, f, g). Immunolabelling of P2X4 throughout was abolished by preabsorbing P2X4 antibody with excess peptide molecules in the controls (Fig. 1i). The expression of P2X4 was evident in the hair cells from E20.5 but was more prominent in the IHCs than OHCs. When compared with the OoC at different rat ages, (E20.5, P4, P8 and P21); Fig. 1c–f), immature IHC at E20.5 expressed P2X4 above the background, but the signal was relatively weak. At P4 and P8, expression of P2X4 was clearly evident in IHCs and OHCs. By P21, a week after the hearing onset, P2X4 exhibited a similar expression pattern to the adult cochlea with strong expression of P2X4 in the IHCs. At P8, P2X4 expression was prominent in IHC and OHC (Fig. 2a, b). Some cells lining the cochlear scala tympani and vestibuli also expressed detectable levels of P2X4 (Fig. 2a, b, arrowheads). It is not possible to identify these cells; however, they appear morphologically very similar to Iba1-expressing macrophages observed in the postnatal mouse cochlea (Kishimoto et al. 2019). To confirm the identity of cells expressing P2X4, two cell-type specific markers were used: myosin VIIa, which is consistently expressed in IHC and OHCs (Jung et al. 2019; Xiong et al. 2019), and Sox2, which is a transcription factor expressed in nuclei of all types of supporting cells (Smeti et al. 2011). P2X4-labelled cells co-expressed myosin VIIa, confirming these to be the IHCs and OHCs (Fig. 2g–g‴), while P2X4 was not observed in cells expressing Sox2 (Fig. 2h–h’’’).

### Polarity of P2X4 subcellular distribution within IHC and OHC in adult rat cochlea

**Fig. 3:**
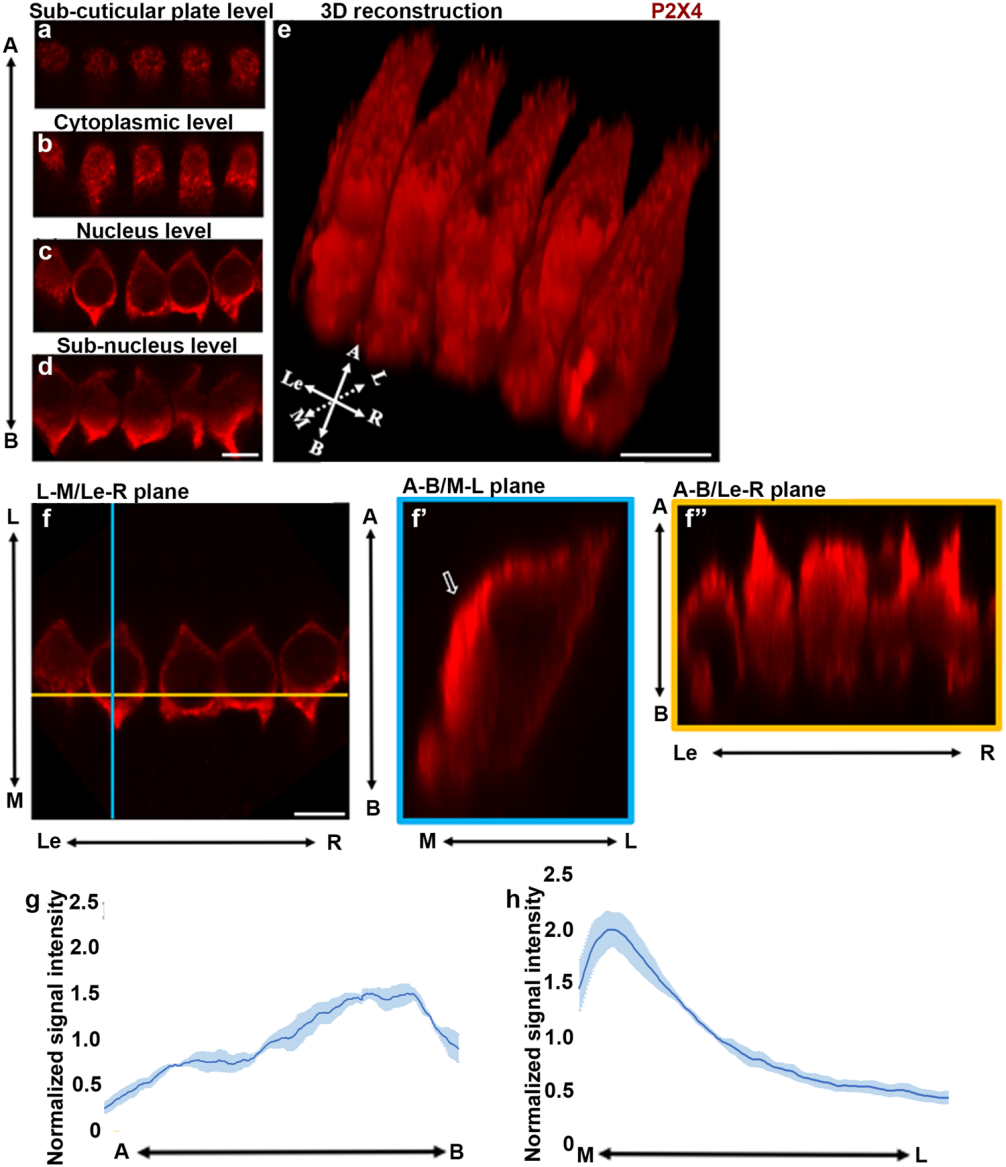
Subcellular distribution of P2X4 in IHC. **a**–**d**) The P2X4 expression in the IHCs compared at different focal planes visualised by optical slicing along the *z* axis. The axis on the left represents the images taken from the apical (A) to the basal (B) region of the cells. **e** The 3D reconstruction of the IHCs. The axis on the left bottom corner represents the cell orientation. **f**–**f″**) The orthogonal views of the IHCs with three planes. The bright P2X4 signal at the medial end of the IHCs. Axis in the figure are: A, apical; B, basal; L, lateral (towards lateral wall side); M, medial (towards modiolus side); Le, left; R, right. Scale bars = 10 μm. **g**, **h** P2X4 normalised signal intensity (*y* axis) from three cochlea along the apical–basal axis (**g**) and medial–lateral axis (**h**)

We next investigated the subcellular localisations of IHCs and OHCs in the adult rat cochlea to correlate the distribution of P2X4 with the distinct functional domains of IHC and OHC. Analysis of Z-stack images of OoC enabled the compartmentalisation of the hair cells into four different subdomains from the apical surface to the basal pole of the cell; subcuticular (Fig. 3a), cytoplasmic (Fig. 3b), nuclear (Fig. 3c) and subnuclear zones (Fig. 3d). Z-stack imaging in combination with three-dimensional (3D) reconstruction showed that the brightest P2X4 labelling was observed along medial/basal aspects of IHC, the surface facing the border cells. At the subcuticular plate level of IHC, P2X4 immunolabelling appeared as bright irregular clusters in the cell cytoplasm immediately underneath the cuticular plate (Fig. 3a). In the supranuclear cytoplasm, between the cuticular plate and nucleus, there were similar clusters of P2X4 immunolabelling; however, these appeared larger and brighter (Fig. 3b). At the nucleus and subnucleus levels, the cytoplasmic immunolabelling for P2X4 appeared brightest and the most abundant (Fig. 3c, d). This pattern is also evident in the 3D re-constructions (Fig. 3e). Orthogonal views of the images were also generated with ImageJ to enable comparison along the left and right (Le-R), medial and lateral (M-L) and the apical and basal (a-b) axes, respectively (Fig. 3f). Orthogonal visualisation confirmed more intense P2X4 immunolabelling along the medial side of the IHCs (Fig. 3f″, open arrow). This corresponds to the large, patchy signal appearance in the 3D re-construction (Fig. 3e). More intense signal was also observed at the apical part of the image (Fig. 3f″). Signal distribution for P2X4 along the apical–basal and medial–lateral axes of the cells was quantified using ImageJ (Supplementary material 3) to confirm visual observations that the P2X4 expression was more concentrated at the basal end of the IHCs (Fig. 3g) and at the medial side of the IHCs (Fig. 3h).

**Fig. 4:**
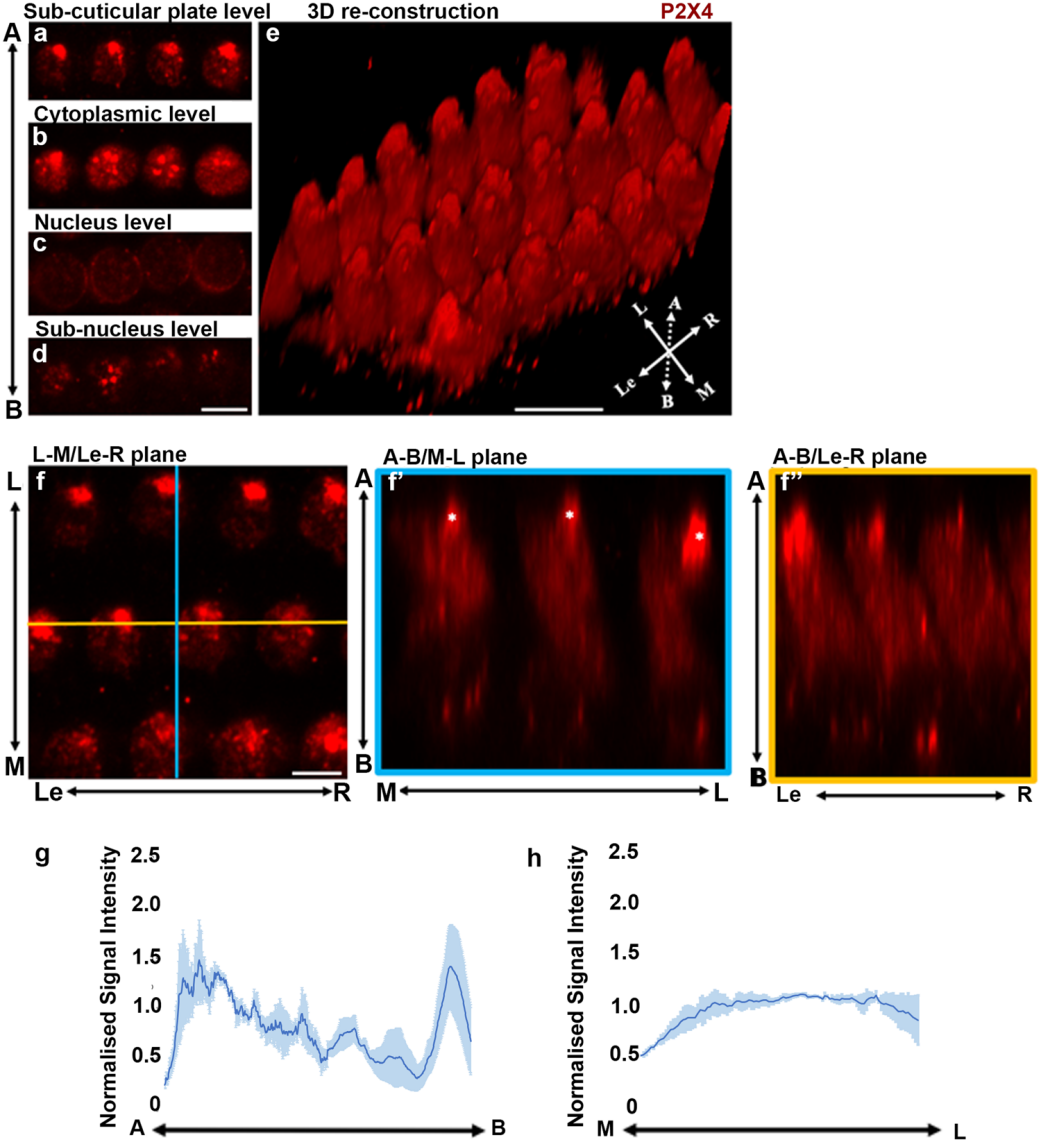
Subcellular distribution of P2X4 in OHC. **a**–**d** The P2X4 expression in the OHCs compared at different focal planes visualised by optical slicing along the *z* axis. The axis on the left represents the images taken from the apical (A) to the basal (B) part of the cells. **e** The 3D reconstruction of the OHCs. The axis on the left bottom corner represents the cell orientation. **f** The orthogonal views of the OHCs with three planes. *The prominent cluster of P2X4 vesicles in the apical–lateral end of the OHCs. Axis in the figure are: A, apical; B, basal; L, lateral (towards lateral wall side); M, medial (towards modiolus side); Le, left; R, right. Scale bars = 10 μm. **g**, **h** P2X4 normalised signal intensity (*y* axis) in three cochleae along the apical–basal axis (**g**) and along medial–lateral axis (**h**)

**Fig. 5:**
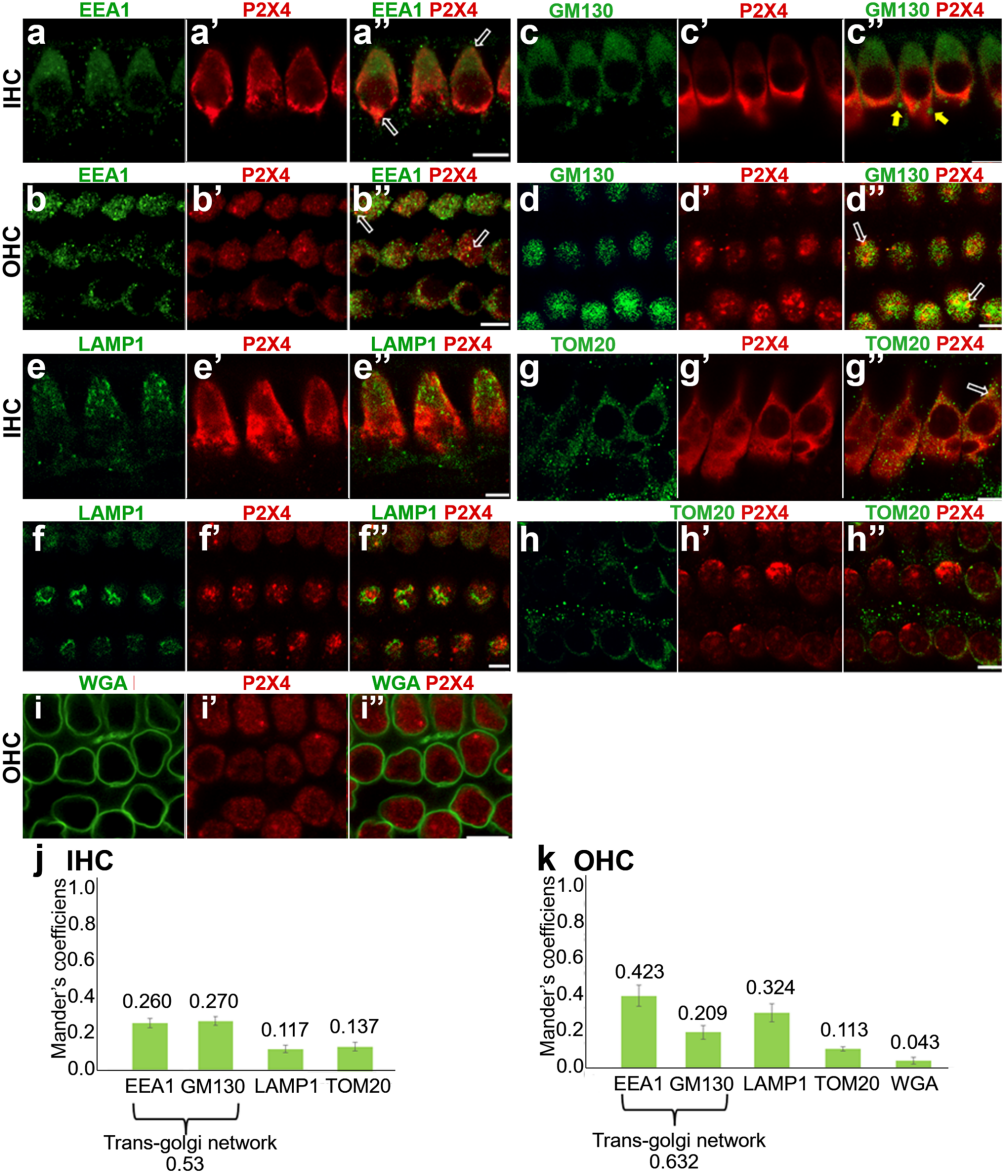
Co-localisation of P2X4 in IHCs and OHCs with organelle markers. **a**–**j** OoC preparation from adult Wistar rats were immunolabelled with an anti-P2X4 antibody (red) and one of the organelle markers (green); anti-EEA-1 (**a**, **b**), anti-LAMP-1(**c**,**d**), anti-GM130 (**e**, **f**), anti-TOM20 (**g**, **h**) and WGA (**i**). High-resolution images were taken at IHC (**a**, **c**, **e**, **f**) and OHC (**b**, **d**, **f**, **h**, **i**). Open arrows indicate a double-positive signal, while closed arrowheads indicate a single-positive marker signal. Scale bars = 10 μm. (**h**, **k**) Mander’s coefficient (value range 0–1.0) has been summarised. *n* = 3 cochleae were examined

Similar analyses in the OHCs (Fig. 4) showed P2X4 immunolabelling at all four levels predominately in the cell cytoplasm; however, the characteristic pattern of P2X4 localisation was quite different from that observed in IHCs. At the subcuticular plate level, the P2X4 expression appeared to be more concentrated than observed in IHC (Fig. 4a). Interestingly, the cluster of P2X4 labelling often appeared immediately underneath the ‘cuticular-free zone’, a small region on the lateral aspect of the cell that does not stain with phalloidin (Fig. 4b). At the cytoplasmic level, regions of P2X4 appeared more scattered, but some medium-sized clusters were observed (Fig. 4b). At the nucleus level, the P2X4 immunoreactivity was less obvious (Fig. 4c), but more intense in the basal subnucleus level of OHC (Fig. 4d). When reconstructed in 3D, a prominent cluster of P2X4 immunolabelling was observed at the apical part of the cytoplasm, and it was not as homogeneously distributed through the whole cell compared with P2X4 immunolabelling in IHC (Fig. 4e). Examined using the orthogonal view, the most intense signal for P2X4 (Fig. 4f′, asterisks), appearing as a prominent cluster, was observed at the lateral side of each cell underneath the Cuticular Plate (CP)-free zone (Fig. 4e). Z-stack images obtained for the OHCs were quantified using ImageJ to confirm that the P2X4 expression was more concentrated at both the apical and the basal end of the OHCs (Fig. 4g). The gradient in OHC was very subtle in the medial to lateral direction (Fig. 4h), compared with the clear trend observed for IHC (Fig. 3g). Additional ‘particle analysis’ of P2X4 also showed that P2X4-labelled particles occupied more areas towards the apical aspect than the basal aspect of the OHC (Supplementary Material 1). The total area occupied by P2X4 immunolabelling was the greatest in the subcuticular zone compared with the three other zones (Fig. 5c). By contrast, the total amount of staining was the highest in the cytoplasmic zone compared with the other three zones (Fig. 5d).

### Localisation of P2X4 to subcellular organelles within IHCs and OHCs.

**Fig. 6:**
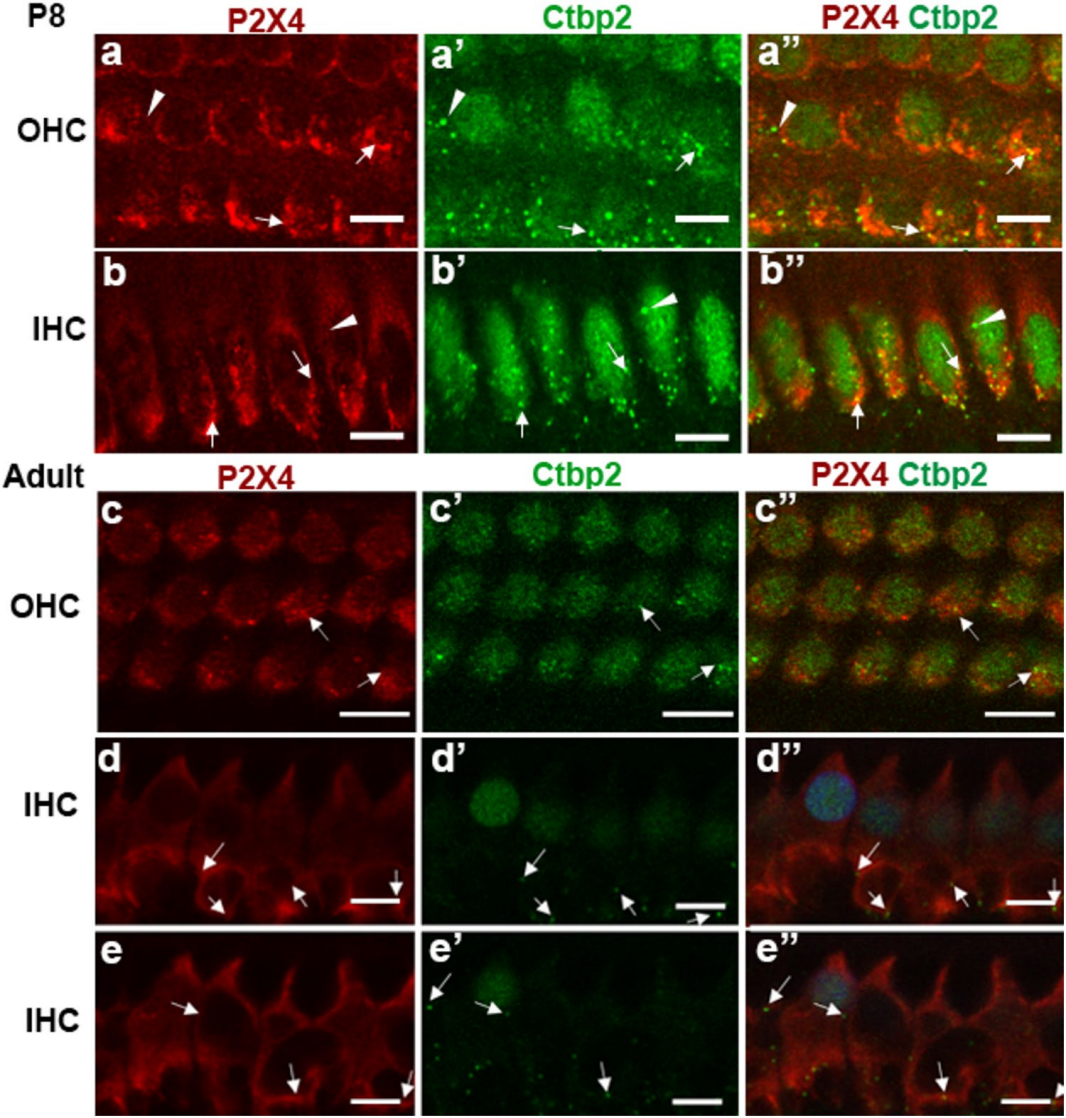
Co-localisation of P2X4 with pre-synaptic membrane marker, anti-Ctbp2 antibody. Wholemount preparation from P8 Wistar rat cochlea (**a**, **b**) and adult rat cochlea (**c**, **e**) was labelled with anti-P2X4 antibody (red) and anti-CtBP2 antibody (green) and imaged with a confocal microscope (60x, Z-stack). Optical sections were taken at the basal planes of OHC (**a**–**a”**, **c**–**c’’**) and IHC (**b**-**b’’**, **d**–**e’’**). White arrows indicates examples of where P2X4 and Ctbp2 immunolabel co-occurred. Scale = 10 μm. Images representative from four cochleae (P8) and five cochleae (adult). DAPI (blue) labelling was included for adult cochlea only

The P2X4 immunostaining was in clusters and appeared to be vesiculated. To determine if these were associated with other membranous intracellular organelles, we looked at co-localisation of P2X4 with endosomes, lysosomes, Golgi bodies and mitochondria using immunohistochemistry (Table 1). Early endosomes are derived from the plasma membrane (Gindhart and Weber 2009) and distinguished from late endosomes and other vesicles by the expression of early endosome antigen 1 (EEA-1) (Patki et al. 1997), including in IHCs and OHCs (Schug et al. 2006). Endosomes and the Golgi apparatus are part of the intracellular protein transportation and recycling pathway. EEA-1 labelling in IHCs had a diffuse appearance, with vesicular labelling more concentrated in the apical part of the cell (Fig. 5a). In OHCs, EEA-1 labelled vesicles appeared throughout (Fig. 5b). There was some co-labelling between EEA-1 and P2X4 (Fig. 5a, b arrows) in both IHC and OHC, with qualitatively more co-occurrence observed in OHC. To quantify the co-localisation of EEA-1 with P2X4, the JACoP plugin (Bolte and Cordelières 2006) in ImageJ was used (see Supplementary Fig. 3). Z-stack images covering either entire OHCs or IHCs were selected for analysis. JACoP quantifies the co-occurrence of P2X4 and EEA-1 as two ‘Mander’s coefficients’ calculated as M1 and M2 coefficients with a value range between 0 and 1.0. M1 represents the proportion of EEA-1 co-localised with P2X4 signal over the total P2X4 signal. M2 represents the proportion of the EEA-1 co-localised with P2X4 over the total signal of EEA-1. The average M1 values for each organelle marker in IHC and OHC are summarised in Fig. 6j, k. Taking the same approach, we analysed the co-occurrence of P2X4 with LAMP-1, GM130, Tom20 and Wheat Germ Agglutinin (WGA). LAMP-1 is a protein found on lysosomes and lysosome-endosome fusion vesicles and is commonly used as a marker for lysosomes (Huotari and Helenius 2011). Lysosomes are distributed throughout the cell in the IHCs and OHCs, but large lysosomes are often found at the apical, lateral side of the cell (Spicer et al. 1999). OHCs have a greater number of lysosomes compared with IHCs (Spicer et al. 1998; Wiwatpanit et al. 2018). LAMP-1 labelling in IHCs had a more diffuse appearance with lower signal levels, and minimally co-occurred with P2X4 (Fig. 6c) where the OHCs had a vesicular appearance (Fig. 6d). There was a clear overlap of the P2X4 immunolabelling and LAMP-1 in OHC (Fig. 6d, Table 2). GM130 is a marker for Golgi matrix protein of 130kDa, which typically targets the cis-component of Golgi (Nakamura et al. 1997). The Golgi apparatus is located mainly around the apical part of the cytoplasm in HCs (Schug et al. 2006; Spicer et al. 1998, 1999). In the rat cochlea, cytoplasmic expression of GM130 was observed in the IHCs and OHCs with vesicular, string-like structures (Fig. 6e, f), consistent with previous reports (Schug et al. 2006). Notably, the co-occurrence of the GM130 and P2X4 in both the IHCs and OHCs was minimal (Fig. 6d, f), 0.117 ± 0.024%, and in the OHCs, 0.27 ± 0.02. TOM20 is a protein expressed on the mitochondrial outer membrane (Balaker et al. 2013) and was used here as the marker for mitochondria. There was some overlap of TOM20 and P2X4 signal in the IHCs (Fig. 6g, arrow). However, there was little co-localisation between P2X4 and TOM20 in both OHCs and IHC (Fig. 6h). TOM20 was co-occurred with P2X4 in the IHCs 0.137 ± 0.025 and in OHCs 0.113 ± 0.012. Finally, WGA is naturally occurring molecule known to bind to glycoproteins found in the cell membrane, and fluorescent conjugates are commonly used as a marker for cell membrane (Emde et al. 2014). The WGA labelled the OHC membrane but did not stain IHC, similar to a previous study (Gil-Loyzaga and Brownell 1988). Therefore, the association with the IHC membrane was inconclusive and therefore not included in this study. We observed the minimal overlap between WGA and P2X4 in the OHCs (Fig. 6I). WGA was co-localised with P2X4 in OHCs 0.043% ± 0.002 (Fig. 6K). Taken together, in IHCs, EEA-1 and GM130 have the highest co-localisation with P2X4 compared with other organelle markers, suggesting cytoplasmic P2X4 were likely associated with endosomes and Golgi apparatus. The co-localisation pattern in OHCs was slightly different from that with IHCs; EEA-1 and LAMP-1 have a higher percentage of co-localisation with P2X4. This suggests that P2X4 associate with endosomes and lysosomes in OHCs (Fig. 6k, l).

**Table 2:** Co-labelling of SGNs with anti-P2X_1_ antibody and anti-peripherin-1 antibody

Co-localisation cell count (SGNs)	Adult (*n* = 4)			P8 (*n* = 4)		
	Apical	Mid	Basal	Apical	Mid	Basal
Total number of SGN counted (*n* = 4 cochlea)	333	410	101	501	480	153
P2X_1_ positive	29	32	11	56	53	22
Peripherin-1 positive	45	41	1	68	53	24
Peripherin-1 and P2X1 positive	29	32	1	56	53	22

Finally, having observed the localisation of P2X4 towards the basal aspect of IHC and OHC, we investigated if any of P2X4 vesicles were located near the presynaptic terminal using anti-Ctbp2 antibodies. Anti-Ctbp2 is raised to recognise RIBEYE in presynaptic ribbons (Kujawa and Liberman 2009; Liberman et al. 2011). A co-labelling experiment conducted in P8 cochlea and adult cochlea showed that some Ctbp2-labelled vesicles on the basal aspect of hair cells appeared to overlap with P2X4 labelling (Fig. 6, arrowheads); other puncta did not overlap, particularly at P8 (Fig. 6 arrowheads). The observation was that P2X4 immunolabelling was more abundant than the Ctbp2 immunolabelling near the membrane.

## Discussion

In this study, we have comprehensively mapped the expression of P2X4 in the Wistar rat cochlea using immunohistochemistry and reported the expression in the IHCs and OHCs of the Wistar rat cochlea for the first time. The minimal expression of P2X4 labelling in the stria vascularis was unexpected, as literature suggests that P2X4 receptors are expressed in the endothelial cells of spiral ligament capillaries in the lateral wall of the guinea pig cochlea (Wu et al. 2011). This discrepancy might occur because a different antibody from Abcam (UK) was used, which is no longer available on the manufacturer website. The expression of P2X4 in sensory HCs was observed uniformly throughout the apical, middle and basal turn of the cochlea (Supplementary Fig. 4). P2X4 immunolocalisation was observed in a small population of cells in the spiral ligament and the spiral ganglia; however, the frequency was very low in the adult cochlea. It is important to note that the P2X4 subunit can form heteromeric channels with P2X1 5, 6 and 7 subunits. P2X4 immunolocalisation in the OoC in our study is distinctively different from what has been reported for P2X1 (Xiang et al. 1999), P2X7 (Nikolic et al. 2003) and P2X2 (Jarlebark et al. 2000; Wang et al. 2003), which might suggest that P2X4 has different roles compared with other isoforms. The most intense P2X4 expression was observed in the IHCs and OHCs, where P2X4 was predominately localised in the cytoplasm with distinct polarity in the subcellular distribution of the receptor protein. Using organelle markers, we show that the majority of cytoplasmic P2X4 was co-localised with vesiculated structures, particularly early endosomes marker EEA1 and Golgi (trans-Golgi network) marker GM130. Additional association with lysosomes occurred in OHCs at higher trend than observed in IHC. There was little evidence of P2X4 expression in the cell plasma membrane, although there was some cooccurrence of P2X4 with Ctbp2 immunoreactivity.

The cytoplasm of IHCs and OHCs are enriched with endosomes (Spicer et al. 1998, 1999). This P2X4 localisation may represent a pool of P2X4_,_ which will become inserted into the membrane under certain conditions, or it reflects continual membrane–cytoplasm cycling. Cytoplasmic P2X4 has been reported in many tissues, including alveolar epithelium and neurons (Bobanovic et al. 2002; Qureshi et al. 2007; Stokes et al. 2017). In the ocular lens, cytoplasmic P2X4 becomes more associated with the cell membrane under osmotic stress (Suzuki-Kerr et al. 2009), supporting the notion that cytoplasmic vesicles containing P2X4 are dynamic. Interestingly, the distribution of cytoplasmic P2X4 showed polarity within the cell. The apical cell domain of both OHCs and IHCs are in contact with potassium-rich endolymphatic fluid, whereas the basal-lateral domain is in contact with sodium-rich perilymph and has the pre-synaptic clefts for synaptic transmission. A large proportion of cytoplasmic P2X4 immunolabelling in the IHCs occurred on the apical and basal ends, in proximity to the synaptic cleft, which may suggest physiological P2X4 roles in regulating pre-synaptic function.

In addition, the cytoplasmic labelling in the IHCs was concentrated adjacent to the medial side of the lateral membrane. The medial side of IHCs is adjacent to the inner border cells, which abundantly express connexin 26 and 30, and while their primary role is communication between supporting cells as gap junctions, they also exist as connexin hemi-channels (Taylor et al. 2012; Zhao et al. 2005). It is interesting to speculate that P2X4 may be activated in a paracrine manner by connexin hemichannel-mediated ATP released from the inner border cells. Such a gradient of P2X4 distribution in the IHCs was not observed in younger animals (P4–P8), suggesting that P2X4 signalling may be established in mature IHCs. Localisation of P2X4 to the synapse has previously been reported in several studies at synaptic terminals in the central nervous system (Lalo and Pankratov 2023). Our observation, which suggest roles for P2X4 in IHC and OHC synapses, is interesting. Previously, other ATP receptors such as P2X1 have been shown to be found at hair cell-SGNs as potential post-synaptic receptors (Nikolic et al. 2001; Xiang et al. 1999). ATP signalling has been suggested to play a role in Type II SGNs as the ‘trauma detector’ in the cochlea which is activated when OHCs are damaged (Liu et al. 2015). Future studies should explore the identity of whether P2X4 is expressed in sensory cells as the pre-synaptic receptor; it would mean that different P2X receptor subtypes play roles with pre- and/or post-synaptic ATP receptors. While it is challenging to functionally discriminate P2X subtypes, it would be of great interest to explore this further in future studies.

OHCs are the other type of sensory epithelial cells in the cochlea; however, they have distinct functional role than IHCs as part of the ‘cochlear amplifier’ by contracting and elongating in response to sound (Pickles 1998). The robust expression of P2X4 was found in OHCs mainly in the cytoplasmic space near the apical membrane and also towards the basal membrane. This was less evident in young animals, suggesting critical roles for P2X4 in more mature OHCs. The large proportion (63.2%) of cytoplasmic P2X4 in OHCs co-occurred with trans-Golgi network similar to the IHCs, and this may represent the dynamic cycling pool of P2X4 receptors moving to and from the plasma membrane. Given the close proximity of vesicular P2X4 to the apical and basal membranes, we may speculate that ATP is released from Deiters cells underneath OHCs which express connexin 26 and 30, proteins capable of forming hemi-channels and gap-junctions (Hosoya et al. 2021; Taylor et al. 2012; Zhao et al. 2005). In addition to the robust basal expression, both qualitative and quantitative analysis showed a robust P2X4 expression at the apical subcuticular level of the OHCs, where they exhibited a very characteristic appearance of ‘plaque’ or ‘cluster’ of vesicles. These were often found immediately underneath the CP-free zone. While only 11.7% of P2X4 co-localised in IHCs with the lysosome marker, a greater proportion (32.4%) of P2X4 co-localised with the lysosome marker in OHCs, showing differences between IHCs and OHCs. One possibility for lysosomal localisation of P2X4 is a part of protein cycling; the late-endosome will fuse with lysosome during protein degradation, some of which may correspond to P2X4 receptors trafficking en route for degradation. Alternatively, P2X4 has been suggested to play a role as a lysosomal ionic channel on the basis of observation in cultured neurons (Murrell-Lagnado 2018; Murrell-Lagnado and Frick 2019). Lysosomal P2X4 receptor activation is influenced by pH within the lysosome lumen in cell culture and induces membrane fusion (Cao et al. 2015). In the cochlea, lysosomal dysfunction has been reported to lead to cellular toxicity in OHCs but not in IHCs (Wiwatpanit et al. 2018). It would be interesting to explore the difference in lysosomal physiology between IHCs and OHCs and how P2X4 in OHC lysosomes may be involved in such a process. Understanding the role of P2X4 will require further investigation into the physiological activation of P2X4, including pharmacological manipulations. P2X4 receptor signalling and its intracellular roles in the cochlea likely contribute to the sensory cell physiology and pathophysiology.

## Supplementary Information

Below is the link to the electronic supplementary material.

Supplementary file1 (DOCX 2136 KB)

## Data Availability

No datasets were generated or analysed during the current study.

## Abbreviations

ADP: Adenosine 5′-diphosphate
ATP: Adenosine 5′-triphosphate
BDNF: Brain-derived neurotrophic factors
Ca^2+^: Calcium ion
dB: Decibels
EP: Endocochlear potential
IHCs: Inner hair cells
JACoP: Just Another Co-localisation Plugin (ImageJ plugin)
K^+^: Potassium ion
kHz: Kilohertz
μM: Micromolar
Mo: Modiolus
OHCs: Outer hair cells
OoC: Organ of Corti
ROI: Region of interest
SGN: Spiral ganglion neuron
SNHL: Sensorineural hearing loss
SV: Stria vascularis
UDP: Uridine diphosphate
UTP: Uridine triphosphate
v/v: Volume per volume
w/v: Weight per volume
WGA: Wheat germ agglutin
WHO: World Health Organization
