# Supplementary material for: Subcellular localization of the P2X4 receptor in sensory hair cells of Wistar rat cochlea
Source: Histochem Cell Biol. 2025 May 20;163(1):54. doi: 10.1007/s00418-025-02386-1 (PMC12092529; doi:10.1007/s00418-025-02386-1)
Supplement: Supplementary file 1 — Supplementary file1 (DOCX 2136 KB) [file 418_2025_2386_MOESM1_ESM.docx]

# Supplementary Information

Journal of Histochemistry and Cell Biology

Subcellular Localization of the P2X_4_ Receptor in Sensory Hair Cells of Wistar Rat Cochlea. Ziyin Huang^1,2,3^, Jacqueline M Ross^4,5^, Shelly CY Lin^1^, Kevin Roy^1^, Prakansha N Kumar^1,2,3^ Srdjan M Vlajkovic^1.3^, Peter R. Thorne^1,2,3^ and Haruna Suzuki-Kerr^1,3^

**Affiliations:**

1. Department of Physiology, The University of Auckland, New Zealand

2. Department of Audiology, The University of Auckland, New Zealand

3. Eisdell Moore Centre, The University of Auckland, New Zealand

4. Department of Anatomy and Medical Imaging, The University of Auckland, New Zealand

5. Biomedical Imaging Research Unit, The University of Auckland, New Zealand

**Corresponding Author:**

Haruna Suzuki-Kerr

h.suzuki-kerr@auckland.ac.nz

**Suplementary Material 1: Particle analyses of P2X4 labeling in OHC.**


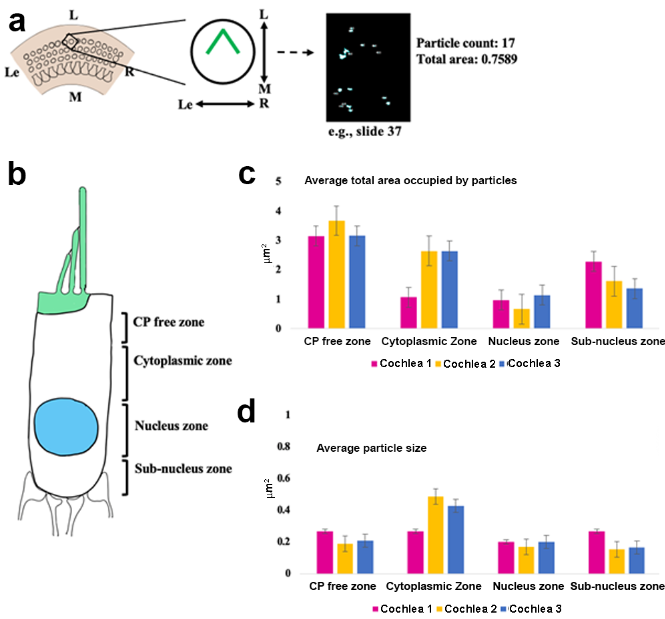


**Supplementary Figure 1. Particle analysis of P2X_4_ immunolabeling in OHCs using ImageJ.** The original z-stack images were imported into Imagej software, and individual channels were separated. To separate vesicular/punctate labelling by thresholding, background subtraction was necessary; images from the channel of interest were duplicated and processed using the Gaussian Blur (radius = 40 pixels), which was used “background” for background subtraction using the “image calculator” function of ImageJ. ROI was selected so that one cell was analysed at a time. Three OHCs and IHCs were analysed per cochlea, and one OHC was selected from each row. Image stack was processed by thresholding into individual binary images, and particle analysis in ImageJ was used to automatically detect particles to measure the parameters “count” and “total area. “Count” represents the total number of particles in each image, and “total area” represents the total area of the particles in each image. The particles’ parameters were set to include any size particle with no limit on their circularity. **(a)** Schematic example of the selection of the cell and the particle analysis example. Each Z-stack of OHCs contains 70-90 optical sections depending on the plane of focus. Each slide in the Z-stack is displayed with particle counts and total area. **(b)** Schematic OHCs with the zone division. **(c)** The summary graph of the P2X_4_ expression average total area occupied by all “particle” estimated by ImageJ plug in. **(d)** Area normalised per particle. Axis in the figure is: [L] lateral (towards lateral wall side) [M] medial (towards modiolus side) [Le] left [R] right. Nine cells from three adult Wistar rat cochleae were subjected to the particle analysis. The results from this analysis were in alignment with our qualitative observation that there were more P2X_4_-labelled vesicles localised to the subcuticular region. This was not because of the difference in average particle area (i.e. size).

**Supplementary Material 2:** **Methodology for determining the relative signal intensity (Figure 3 and Figure 4 of the main article).**
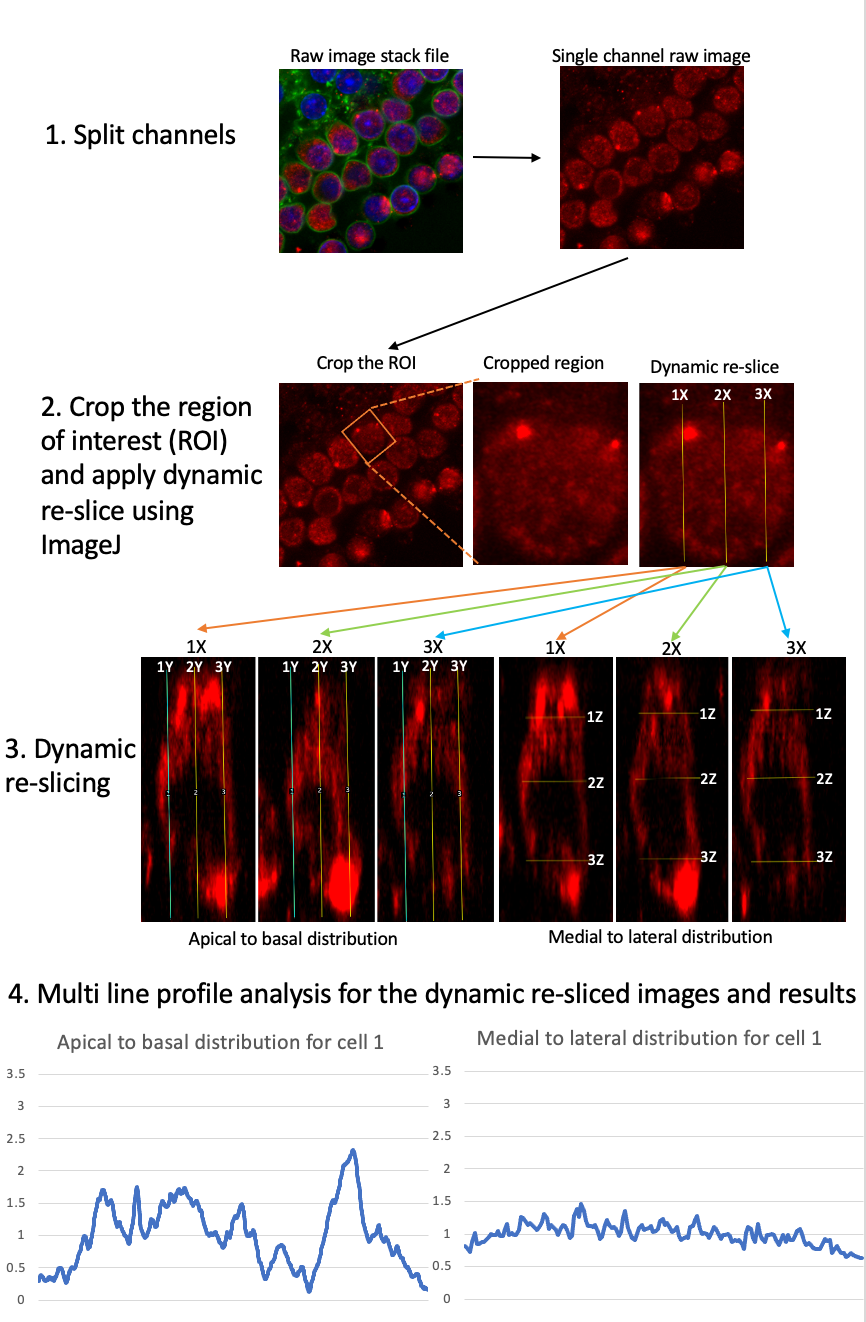


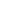


**Supplementary Figure 2. Flow chart showing steps of line plot profile analysis with ImageJ. (1)** ImageJ “Line plot profile” analysis was performed to quantify the intensity of immunofluorescent signals. Raw Z-stack images were imported into ImageJ and channels split. **(2)** The region of interest (ROI) was selected around one cell to analyse one cell at a time. **(3)** Relative signal intensity were quantified as 9 different line profiles within the same cell. This consists of 3 parallel line profiles taken at 3 different positions (e,g, 1X, 2X, 3X) shown in the image. The 2X measurement positions were designed to go through the middle of the nucleus. The 1X and 3X measurement position are determined by the 2X measurement position where both left and right are 3 μm from the middle measurement position. After reconstructing the lateral view of the selected cell, 3 line profiles for apical to basal analysis were generated (1Y, 2Y, 3Y) which follows the same method where the 2Y passes through the middle of the nucleus, where 1Y and 3Y are 3 μm from 2Y laterally (step 3). The medial and lateral line profiles were similar and 3-line plot profile positions (1Z, 2Z, 3Z) were generated. 2Z was the half point of the lateral viewed cell, where 1Z and 3Z were 10 microns from 2Z. Taken together, these steps define nine distinct lines across the apical-basal and the medial-lateral axis of the cell (3). (4) At each of these 9 lines, the “line profile plot” function of ImageJ was used to measure intensity from apical to basal as well as medial to lateral. For each point, the “line profile plot” function quantifies the greyscale pixel value to produce about 900 data points along the apical to basal axis and about 400 data points along the medial to lateral axis. For each cell, the average grey-scale value was calculated from all line profiles, and this value was used to normalise the average line profile.

**Supplementary material 3: Co-localization analysis**


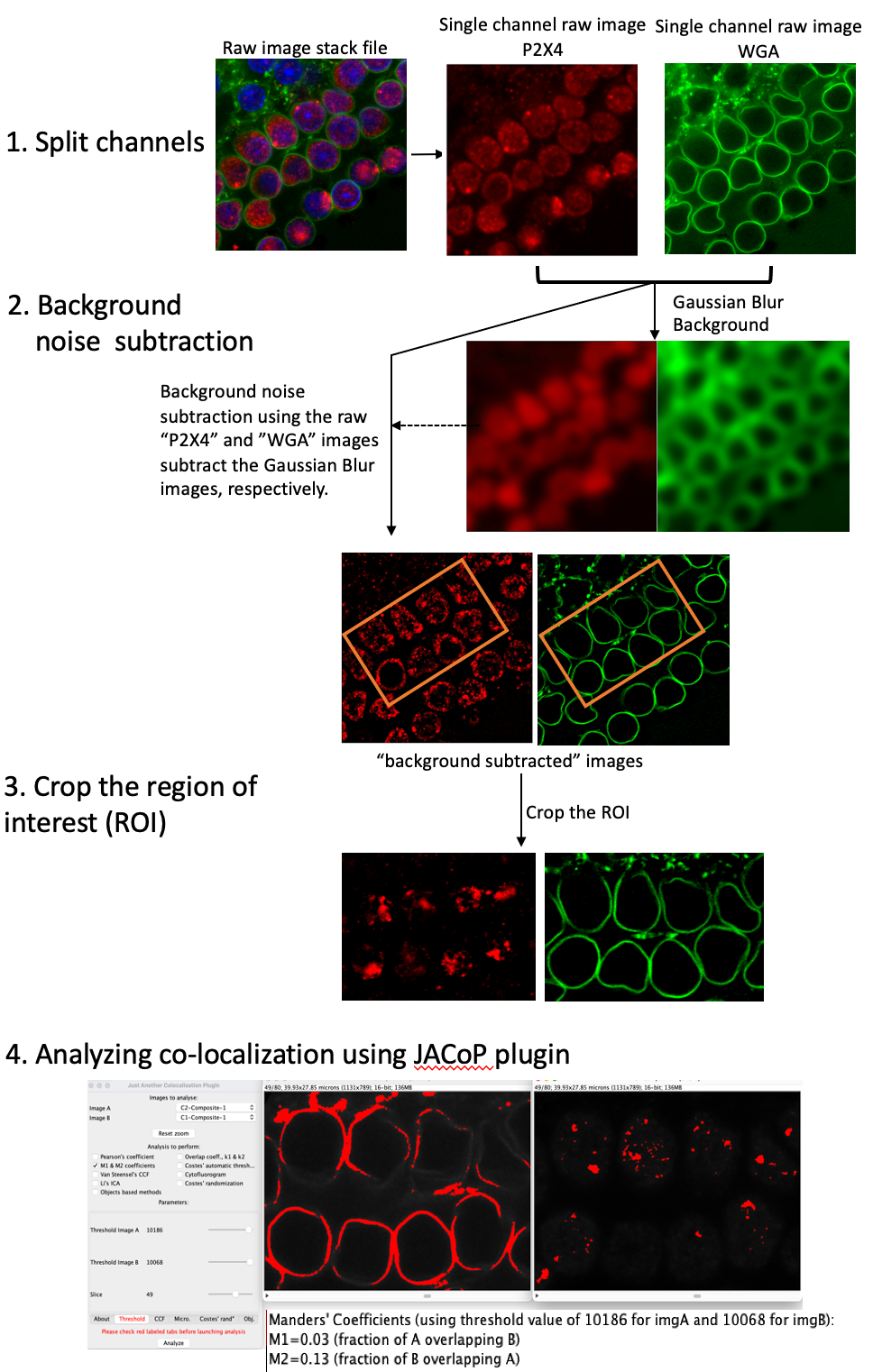


**Supplementary Figure 3. Co-localization analysis steps.** **(1)** Raw Z-stack images of IHCs and OHCs were taken as z-stack. **(2)** As a part of the background subtraction step, images from the channel of interest were duplicated and processed using the Gaussian Blur (radius = 40 pixels) to remove noise and background signals. These blurred images were used as “background”. This was then subtracted from the original image by the “image calculator” function of ImageJ to produce the final “background-subtracted images”. **(3)** The regions of interest were selected to include as many complete cells as possible and exclude signals from adjacent cells and background signals. The typical region of interest contained 8 cells. **(4)** Processed images were used on JACoP to automatically calculate Manders’ colocalization coefficients as the primary output (Dunn, Kamocka, & McDonald, 2011). The results were displayed as M1 and M2. Manders’ colocalization coefficient analysis has an advantage over other methods in that this method can be used reliably even when signals from two channels have different levels of intensity/distribution, and also when the immunolabeling is sparse and the image contains a lot of black/empty space within the field of view (Bolte & Cordelières, 2006). The results of the Manders’ colocalization coefficient, M1 and M2, are given as a value between 0 and 1.0. Colocalization analyses were conducted for each subcellular marker separately with 3 cochleae for each marker, and the mean and standard error of the mean (SEM) were calculated.

**Supplementary Figure 4.**


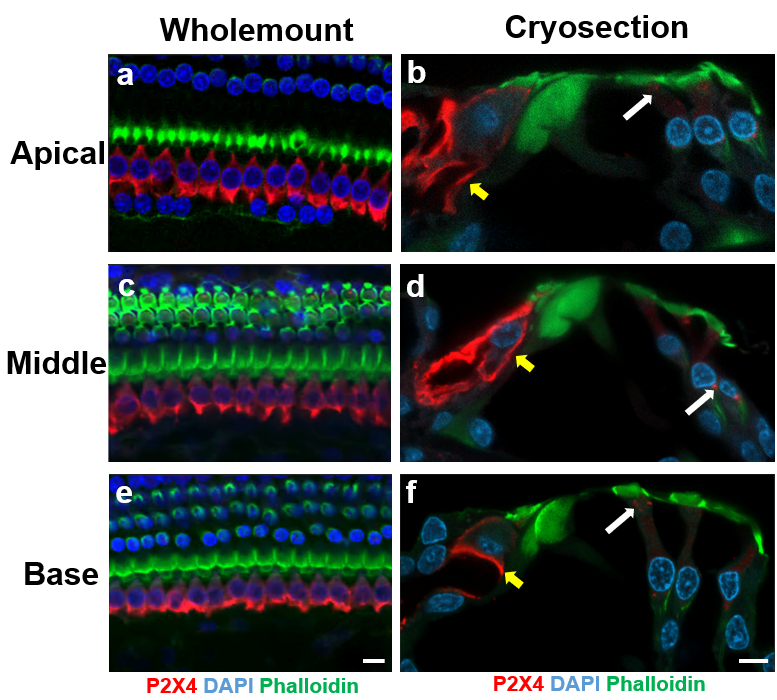


OoC Wholemounts (a, c, e) and 20μm thick cryosections (b, d, f) were prepared from adult rat cochlea within the apical (a & b), middle (c & d) and base (e & f) and labelled with anti-P2X_4_ antibody (*red*), phalloidin (*green*) and DAPI (*blue*). n=3 cochleae were examined. Scale bars = 10 μm. Expression of P2X_4_ in IHC and OHC was observed irrespective of different locations within the cochlea.

**References:**

Bolte, S., & Cordelières, F. P. (2006). A guided tour into subcellular colocalization analysis in light microscopy. *Journal of microscopy, 224*(3), 213-232.

Dunn, K. W., Kamocka, M. M., & McDonald, J. H. (2011). A practical guide to evaluating colocalization in biological microscopy. *American Journal of Physiology-Cell Physiology, 300*(4), C723-C742.
